# Genome sequences of cluster GA phages Phonegingi and Dropshot

**DOI:** 10.1128/MRA.00918-23

**Published:** 2023-11-28

**Authors:** Natalie McLean, Tabitha Jackson, Yajat Govardhan, Aditi Dharanendiran, Caden Bost, Frederick Brown, Benjamin Cowen, Jenna Deselem, Nico Parkhurst, Jacob Stewart, Justin Leonard, D. Parks Collins

**Affiliations:** 1 Department of Natural Sciences, Mitchell Community College, Statesville, North Carolina, USA; Portland State University, Portland, Oregon, USA

**Keywords:** genome announcement, bacteriophage

## Abstract

Bacteriophages Phonegingi and Dropshot were isolated from soil in North Carolina using the host *Microbacterium foliorum*. Both phages have siphovirus morphologies. Based on gene content similarity to one another and to other actinobacteriophages, both phages are assigned to phage cluster GA.

## ANNOUNCEMENT

**Fig 1:**
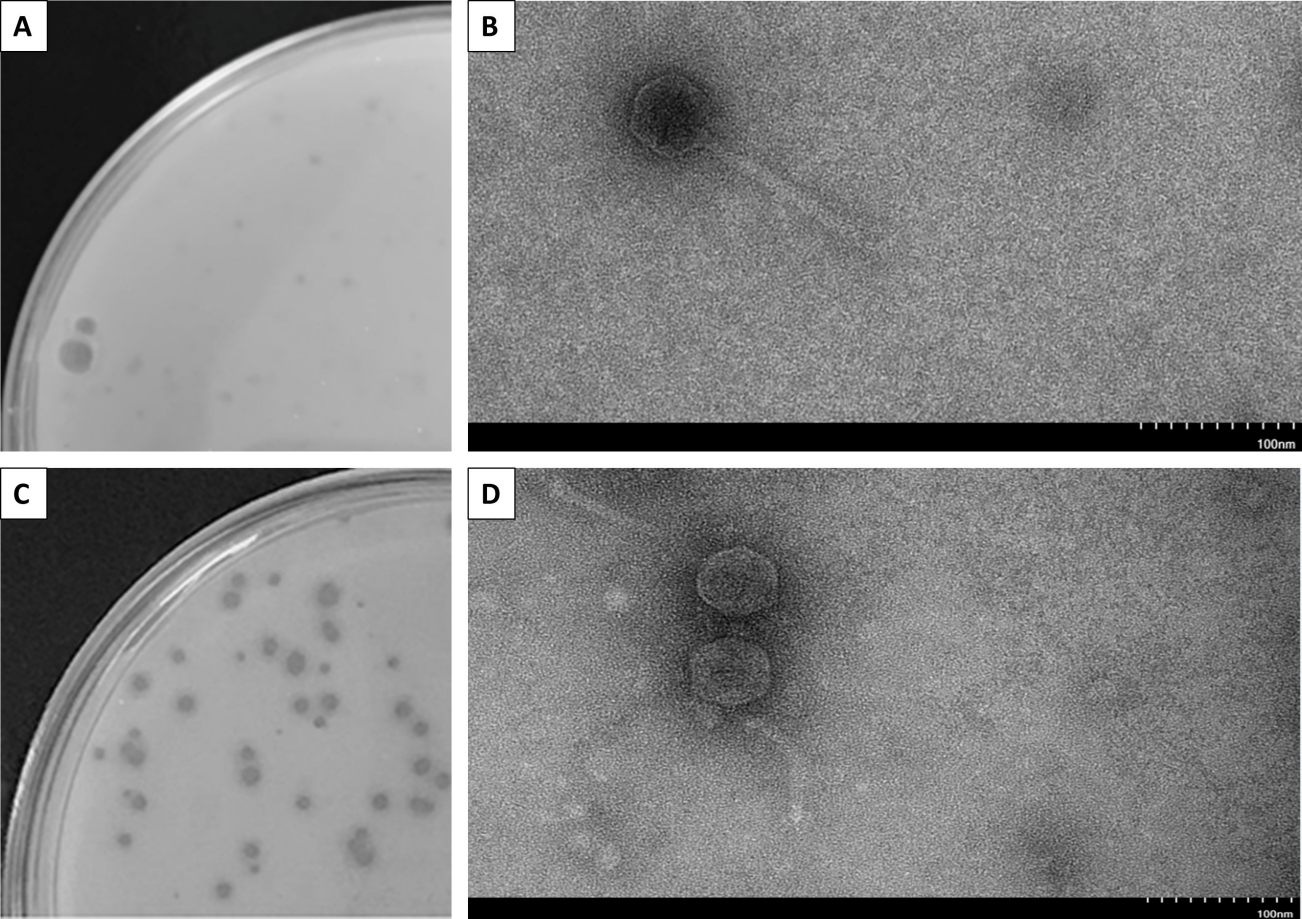
Plaques assays reveal both Dropshot (**A**) and Phonegingi (**C**) to form clear plaques. Negative-stain (1% uranyl acetate) transmission electron microscopy (Hitachi HT7800 120-kV TEM) of Dropshot (**B**) and Phonegingi (**D**) reveal siphovirus morphologies. Scale bar = 100 nm.

Here, we report on the characteristics of two actinobacteriophages, Phonegingi and Dropshot, that were isolated from soil samples using *Microbacterium foliorum* NRRL 24–224 (1). First, soil samples were suspended in peptone-yeast extract-calcium (PYCa) liquid medium before the suspension was spun at 2,000 × *g* for 10 min and the resulting supernatant was filtered (0.22-μm pore size). An aliquot of the filtrate was then plated in PYCa top agar with *M. foliorum*, and the plates incubated at 30°C. After 48 hours, plaques were observed for both soil samples. An individual plaque for each soil sample was selected, and the phage was purified through four rounds of plating. Negative stain transmission electron microscopy of lysates prepared for each phage revealed Siphoviradae morphology for both (Fig. 1). Plaque and particle characteristics are provided in Table 1.

**TABLE 1:** Isolation and sequencing parameters, and particle and genome characteristics of Phonegingi and Dropshot

Phage	Phonegingi	Dropshot
Soil type	Red clay	Mulch
Soil collection site coordinates	35.82831 N, 81.43249 W	35.8677 N, 80.84384 W
Plaque size (nm)	2–3 (*n* = 10)	0.5–5.0 (*n* = 10)
Capsid diameter (nm)	50 (*n* = 1)	40 (*n* = 1)
Tail length (nm)	110 (*n* = 1)	83 (*n* = 1)
Number of sequencing reads	682,088	364,247
Sequencing coverage (*X*)	2,516	1,383
Genome size (bp)	39,364	38,522
GC content (%)	67.6	68
Genome end type	Circularly permuted	Circularly permuted
Cluster	GA	GA
Number of putative protein-coding genes	66	65

DNA was isolated from lysates of Phonegingi and Dropshot using the Wizard DNA cleanup kit (Promega), prepared for sequencing using the NEB Ultra-II Library kit (New England Biolabs), and then sequenced on an Illumina MiSeq instrument. Raw reads (150-base single end) were assembled and verified for completeness using Newbler v.2.6 and Consed v.29, respectively (2, 3). Sequencing parameters and genome characteristics are described in Table 1. Both phage genomes are highly similar, sharing 92% nucleotide identity (4) and 92.5% gene content similarity (GCS), the latter determined using the GCS tool at the actinobacteriophage database (5). Both phages were assigned to actinobacteriophage cluster GA that, to date, consists of only five other phages (6). A BLASTn (4) comparison of Dropshot and Phonegingi against the actinobacteriophage database revealed several short genomics segments of 50–350 bp that share over 75% nucleotide identity with the genomes of cluster EC and EJ phages, highlighting the well-established mosaic nature of bacteriophage genomes (7).

Both phage genomes were annotated using the genome annotation tool PECAAN v.1.0 (discover.kbrinsgd.org). GeneMark v.2.5 (8), BLASTp (4), HHPred (9), TMHMM (10), Starterator v.1.0.1 (http://phages.wustl.edu/starterator/), and Phamerator (11) were used to determine functional assignments. Aragorn v.1.2.41 (12) and tRNAscanSE v.2.0 (13) were used to identify tRNAs. This annotation process identified 68 and 66 putative genes in Phonegingi and Dropshot, respectively, of which approximately half of the genes could be assigned functions. They include structure and assembly functions encoded by genes within the left half of the genomes and lysis functions (a putative endolysin and holin) encoded by genes at the center of the genomes. At the right half of the genomes are genes encoding DNA metabolism functions, including genes with DNA-binding domains (e.g., single-stranded DNA-binding protein) and nuclease domains (e.g., RevC-like resolvase, RecE-like exonuclease, and an HNH endonuclease). As with other cluster GA phages, no integrase or repressor functions could be identified in either genome, suggesting that phages in this cluster are unlikely to establish lysogeny.

## Data Availability

Phonegingi is available at GenBank under accession no. OR475255 and Sequence Read Archive (SRA) accession no. SRX20165778. Dropshot is available at GenBank under accession no. OR475279 and SRA accession no. SRX20165791.
